# Differential Metastatic Patterns and Prognostic Value of Systemic Inflammation Scores in Anaplastic Lymphoma Kinase Rearranged Versus Anaplastic Lymphoma Kinase Negative Non-Small Cell Lung Cancer

**DOI:** 10.3390/cancers18030501

**Published:** 2026-02-03

**Authors:** Melek Özdemir, Gamze Gököz Doğu, Burcu Yapar Taşköylü, Arzu Yaren, Serkan Değirmencioğlu, Atike Gökçen Demiray, Ferda Bir, Bensu Selbest Altay, Burçin Çakan Demirel, Tolga Doğan, Semra Taş, Taliha Güçlü Kantar, Ömer Acar

**Affiliations:** 1 Department of Medical Oncology, Denizli State Hospital, Denizli 20100, Turkey; dr_tolgadogan94@yahoo.com (T.D.); talihaguclu@hotmail.com (T.G.K.); 2 Department of Medical Oncology, Pamukkale University Faculty of Medicine, Denizli 20070, Turkey; ggd2882@gmail.com (G.G.D.); drburcuyapar@gmail.com (B.Y.T.); arzu_yaren@yahoo.com (A.Y.); gokcenakaslan@gmail.com (A.G.D.); semratasdr@gmail.com (S.T.); 3 Medical Oncology, Denipol Hastanesi, Denizli 20010, Turkey; drserkandeg@hotmail.com; 4 Department of Pathology, Pamukkale University Faculty of Medicine, Denizli 20070, Turkey; fbir@pau.edu.tr; 5 Internal Medicine, Denizli Devlet Hastanesi, Denizli 20010, Turkey; drbensuselbest@gmail.com; 6 Medical Oncology, Bağcılar Eğitim ve Araştırma Hastanesi, Istanbul 34200, Turkey; brcn_ckn@hotmail.com; 7 Department of Medical Oncology, Mardin Training and Research Hospital, Mardin 47100, Turkey; dracaromer@gmail.com

**Keywords:** non-small cell lung cancer, ALK rearrangement, systemic inflammation, brain metastasis, liver metastasis, overall survival

## Abstract

**Background**: Non-small cell lung cancer (NSCLC) is the most prevalent form of malignancy and the leading cause of cancer-related fatalities. In clinical practice, metastatic sites are identified on a case-by-case basis. ALK rearrangements are detected in 3–5% of NSCLC cases and are known to have a tendency (tropism) to metastasize to the brain. **Methods**: Data from 81 ALK-positive and 91 ALK-negative metastatic NSCLC patients were retrospectively analyzed. Systemic markers, including HALP score, NLR, PLR, LMR, and LDH, were calculated from blood tests at the time of metastasis. Optimal cut-off values were determined using ROC analysis. Survival outcomes and prognostic factors were assessed using Kaplan–Meier and Cox regression analyses. **Results**: ALK-positive patients were significantly associated with female gender (*p* = 0.002), non-smoking status (*p* = 0.001), adenocarcinoma histology (*p* = 0.001), and a higher incidence of brain metastases (*p* = 0.001). In univariate analysis, age, time to metastasis, liver metastasis, and NLR were prognostic for survival. Crucially, multivariate analysis identified liver metastasis as an independent predictor of poor prognosis (HR = 1.618; 95% CI: 1.050–2.494; *p* = 0.029), indicating a 61.8% increased risk of death or progression. While inflammation markers (NLR, HALP, PLR, LMR) did not predict metastasis to specific sites, elevated LDH levels were significantly associated with liver metastasis (*p* = 0.007). **Conclusion**: ALK-positive NSCLC demonstrates a marked CNS tropism; however, liver metastasis remains a more critical adverse prognostic factor than brain metastasis in real-world settings. While routine inflammation markers showed limited utility in predicting site-specific metastasis, LDH levels correlated significantly with liver involvement. Aggressive management strategies are warranted for ALK-positive patients presenting with liver metastases.

## Simple Summary

Lung cancer is the foremost cause of cancer related fatalities on a global scale. A specific genetic subtype of lung cancer, designated as ALK positive, is characterised by its propensity to metastasise to the brain with high frequency. However, predicting patient outcomes in real-world settings remains a challenging endeavour. The objective of this study was to analyse the disparities between patients with and without this genetic alteration, with a particular focus on the dissemination of cancer and the potential of routine blood tests to predict these patterns. It was established that, while ALK positive patients do exhibit an elevated risk of brain metastasis, metastasis to the liver is a more significant predictor of poor survival. In addition, a standard blood test (LDH) was found to be significantly linked to liver involvement. These findings are of significance as they underscore the necessity for medical professionals to adopt a proactive approach in the management of liver metastases. The utilisation of simple blood tests holds immense potential in the enhanced monitoring of high risk patients, thereby facilitating the development of more personalised treatment strategies.

## 1. Introduction

According to data provided by the World Health Organization (WHO), non-small cell lung cancer (NSCLC) is the most prevalent form of malignancy and the foremost cause of cancer-related fatalities. In order to predict optimal treatment decisions and survival, histopathological and molecular subclassification is necessary [1,2]. The most prevalent pathological subtype of NSCLC cases is adenocarcinoma [3]. Despite the fact that molecular tests are performed on the histopathology of all NSCLC in many centers, the most commonly preferred tests for lung adenocarcinoma—including those for epidermal growth factor receptor (EGFR), anaplastic lymphoma kinase (ALK) rearrangement, ROS proto-oncogene 1 (ROS1), and programmed death ligand 1 (PDL1)—must be performed reflexively [4].

Chromosomal rearrangements of the tyrosine kinase ALK have been detected in 3 to 5 percent of NSCLC cases [5,6]. The most prevalent ALK rearrangement is the EML4-ALK (echinoderm microtubule-associated protein-like 4) fusion oncogene. This results in continuous activation of the tyrosine kinase pathway which, in turn, leads to cell proliferation [7]. While the majority of patients diagnosed with ALK positivity are young and have never smoked, this is not a prerequisite [8]. ALK gene rearrangements can be detected in plasma or tumor tissue. Preferred methods for detecting ALK positivity include immunohistochemistry (IHC), fluorescence in situ hybridization (FISH), next-generation sequencing (NGS), and reverse transcription polymerase chain reaction (PCR) [9,10].

ALK inhibitors are recommended for the treatment of tumors harboring this genetic alteration. A phase III study demonstrated the superiority of crizotinib, a first-generation tyrosine kinase inhibitor (TKI), over chemotherapy, showing an increase in PFS and response rate. However, no OS benefit was identified due to cross-over (HR: 0.76; 95% CI: 0.55–1.05) [11]. A meta-analysis evaluating subsequent studies comparing second-generation ALK TKIs (alectinib, brigatinib, ensartinib) with crizotinib demonstrated an OS benefit for ALK inhibitors (HR: 0.84; 95% CI: 0.72–0.97) [12]. In the event of progression following TKI use, the preferred chemotherapy regimen may be a pemetrexed-based treatment, as per the IMPower150 study [13]. Alternatively, if immunotherapy is favored, a combination of chemotherapy and immunotherapy may be recommended. The combination of TKI and immunotherapy is not recommended due to the potential for fatal toxicity [14]. In the CROWN study, the best survival outcomes were observed with first-line lorlatinib. The five-year PFS rate was 60% in the lorlatinib arm compared to 8% in the crizotinib arm. Demonstrated improvements in survival outcomes have increased the importance of targeted therapies [15,16,17,18].

The identification of distinct metastasis sites has been correlated with the various subtypes of NSCLC. The cancer cell (“seed”) settles in the metastatic tissue as a result of molecular-level interactions between the target tissue’s microenvironment (“soil”) and the cancer cell, rather than randomly. The initial hypothesis that sought to elucidate the mechanisms underlying metastasis (the Seed and Soil hypothesis) was published by Stephen Paget in 1889. In consideration of the results obtained from the autopsy series, it was hypothesized that the increased prevalence of bone metastasis in breast cancer patients could be attributable to the selective settlement of cancer cells in tissue that is compatible with their own. The hypothesis that cancer cells settle in a tissue when they encounter a suitable microenvironment, regardless of blood flow direction, was proposed [19].

Another hypothesis that merits consideration is James Ewing’s “Mechanical-Hemodynamic Theory.” This hypothesis posits that metastasis is contingent on blood flow and vascularization [20]. In contemporary discourse, it is posited that while both theories possess valid points, they are also encumbered by shortcomings. The growth of cancer cells (Ewing) that reach an organ via blood flow is possible if the microenvironment is conducive to this process (Paget). The survival of metastatic cells depends on the presence of a pre-metastatic niche, a fertile environment conducive to their proliferation. This dynamic interaction is hypothesized to result from molecular-level signaling between the seed and the soil [21].

Despite recent advances in molecularly targeted and systemic therapies for NSCLC, the development of brain metastasis is common (organotropism). This is associated with a high mortality rate and poor prognosis. De novo or metachronous brain metastases are observed in 25–40% of ALK-positive NSCLC patients, compared to 20% of all NSCLC patients. Consequently, the elevated incidence of concomitant brain metastases in ALK-positive patients, in conjunction with early diagnosis and timely treatment planning, has been shown to significantly contribute to survival [22].

Phase 3 studies (CROWN [16], ALEX [23]) analyzed ALK-positive NSCLC patients to examine SSS penetration and treatment response; however, the potential to draw conclusions through a comparison of the prognostic impact of other metastatic sites (contralateral lung, bone, liver, and adrenal) in these patient groups with NSCLC patients without driver mutations was unclear. At this juncture, real-world data (RWD) was deemed to be a valuable asset.

A body of research within the relevant literature has concluded that the inflammatory response to metastasis, proliferation, and invasion (which are fundamental characteristics of tumor cells) is important in the process by which tumors grow [24,25]. Cytokines, which are secreted by activated blood cells during the inflammatory response, have been shown to affect tumor cells. In numerous studies [26], an array of systemic, inflammatory, and nutritional markers has been pinpointed in this context.

The analysis of prognostic markers was planned to evaluate the combination rather than a single blood parameter. This was to be achieved by calculating and entering patients’ hematological and biochemical blood results into formulas. For this purpose, the following markers were considered, as they have been shown to be prognostic in patients with NSCLC in the literature: the HALP score (hemoglobin, albumin, lymphocyte, and platelet) [27]; the neutrophil-to-lymphocyte ratio (NLR) [28]; the platelet-to-lymphocyte ratio (PLR) [28]; the lymphocyte-to-monocyte ratio (LMR) [29]; and the lactate dehydrogenase (LDH) level [30]. A growing body of research has emerged in the field of systemic inflammatory response and nutritional status analysis, aiming to predict prognosis and survival.

The objective of this study was to analyze the effect of metastasis patterns and sites (bone, central nervous system, liver, contralateral lung, and adrenal glands) on survival in metastatic ALK-positive NSCLC patients followed at our clinic, compared with an ALK-negative NSCLC cohort. In both groups, blood tests recorded at the time of metastasis were utilized to calculate systemic inflammation and nutrition markers (HALP score (hemoglobin, albumin, lymphocyte, and platelet); the neutrophil-to-lymphocyte ratio (NLR); the platelet-to-lymphocyte ratio (PLR); the lymphocyte-to-monocyte ratio (LMR); and lactate dehydrogenase (LDH). The effects of these factors on the metastatic pattern and prognosis were analyzed. The presentation comprises RWD from a control group that does not include driver mutations.

## 2. Materials and Methods

### 2.1. Data Collection and Patient Characteristics

As indicated by data from the World Health Organization, NSCLC is the most prevalent malignancy responsible for cancer-related fatalities. Despite numerous hypotheses proposed to explain the varied metastasis sites and the metastasis process in NSCLC, unresolved issues remain. In the classification of NSCLC by molecular subtypes, patients without driver mutations (i.e., the control group) were compared with the study group with confirmed ALK positivity (Ventana BenchMark XT automated stainer (Ventana Medical Systems, Inc., Tucson, AZ, USA)). The effect of ALK positivity on metastasis site and the prognostic impact of these metastases on survival were analyzed using RDW to determine outcomes compared with NSCLC patients without driver mutations. In this single-center retrospective cohort study, data from patients diagnosed with advanced-stage NSCLC and subsequently followed up at the medical oncology clinic between January 2015 and June 2024 were analyzed. Ethical committee approval was obtained for this study (date: 10 February 2024; number: E-60116787-020-590646).

### 2.2. Inclusion and Exclusion Criteria for the Study

***Inclusion Criteria:*** The following criteria were established with regard to the inclusion and exclusion of subjects in the present study. All patients diagnosed with NSCLC who were under the care of the medical oncology clinic between the specified dates (January 2015 and June 2024) and who met the exclusion criteria were included in the study. Patients were cytologically confirmed to have advanced-stage NSCLC and were 18 years or older. Patients with complete Positron Emission Tomography (PET), brain magnetic resonance imaging (MRI), and systemic hematological biochemical scans at the time of diagnosis and during follow-ups were included in the study.

**Exclusion Criteria**

- Unknown ALK status;
- The presence of another defined driver mutation;
- The diagnosis of a second malignancy;
- A history of chronic rheumatic disease;
- Patients with missing data.

The clinical and demographic characteristics of the patients were retrospectively recorded from medical oncology patient files and hospital laboratory and imaging system records. A comprehensive dataset was collated, incorporating patient age at diagnosis, gender, smoking status, stage at diagnosis, site of metastasis (brain, liver, bone, pleura/pericardium, adrenal gland, and lymph node), systemic treatments administered, treatment responses, and survival data (median overall survival (mOS) and progression-free survival (PFS)). mOS is defined as the time from diagnosis to death or the last follow-up date. PFS is defined as the time from diagnosis to the first progression of the disease.

**Figure 1:**
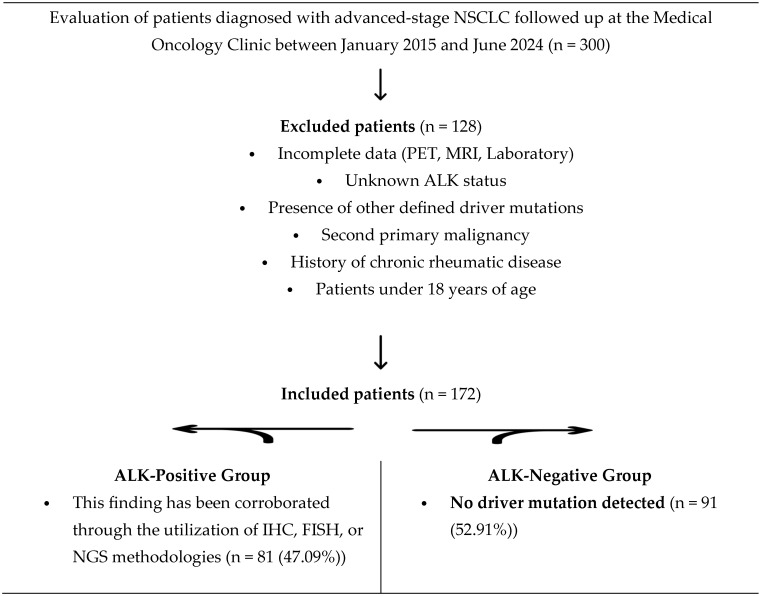
**A flow diagram of the study population selection process.** Over the period from January 2015 to June 2024, 300 patients diagnosed with advanced-stage NSCLC were assessed for eligibility. Following the exclusion of 128 patients based on predetermined criteria, including chronic rheumatic diseases, the presence of other driver mutations, and missing clinical data, a total of 172 patients were included in the final analysis. Patients were categorized into two groups: an ALK-positive study group (n = 81) and a driver-mutation-negative control group (n = 91). Abbreviations: NSCLC: non-small cell lung cancer, ALK: anaplastic lymphoma kinase, IHC: immunohistochemistry, FISH: fluorescence in situ hybridization, NGS: next-generation sequencing.

Laboratory data recorded at the time of metastasis were used to calculate systemic inflammation markers—including the HALP score (hemoglobin, albumin, lymphocyte, and platelet), neutrophil/lymphocyte ratio (NLR), platelet/lymphocyte ratio (PLR), and lymphocyte/monocyte ratio (LMR)—using formulas obtained from the literature. Receiver Operating Characteristic (ROC) analysis was performed to determine the cut-off values for prognostic markers. The effect of ALK status on metastasis sites in NSCLC patients at the time of metastasis diagnosis was planned to be compared as the primary endpoint. The effects of ALK positivity and metastasis site on survival were analyzed as secondary endpoints. The study comprised 81 patients with ALK + NSCLC in the study group and 91 patients with NSCLC without driver mutations in the control group (Figure 1).

### 2.3. The Purposes and Calculation of Prognostic Markers

HALP (hemoglobin, albumin, lymphocyte, and platelet) score: This is a new-generation prognostic marker that quantifies a patient’s nutritional and inflammatory status using a single formula. It is calculated using the formula “((hemoglobin × albumin × lymphocyte)/platelet)” [27].

Lactate dehydrogenase (LDH): This is one of the most established and reliable negative prognostic biomarkers in NSCLC. It is not only a “cell breakdown product,” but also reflects the tumor’s biological behavior [28].

NLR (neutrophil/lymphocyte ratio): This ratio is calculated by dividing the neutrophil count by the lymphocyte count. It is a prognostically significant marker, defined in the literature as indicating an inflammatory response [29].

PLR (platelet/lymphocyte ratio): This ratio reflects the systemic inflammatory status. This prognostic index is derived from the ratio of the absolute platelet count to the lymphocyte count [30].

LMR (lymphocyte/monocyte ratio): This ratio is calculated as the absolute lymphocyte count divided by the monocyte count. This metric serves as a prognostic marker of inflammation [31].

### 2.4. Statistical Analysis

Descriptive data are summarized using means, standard deviations, medians, and minimum–maximum values for continuous variables, while categorical variables are expressed as frequencies and percentages. The distribution normality of numerical variables was assessed via the Shapiro–Wilk test. For comparisons between these variables and categorical groups, the Mann–Whitney U test was applied. Differences among categorical variables were evaluated using the Chi-square test. Survival outcomes were analyzed using the Kaplan–Meier method. To determine the optimal cut-off values for the inflammatory markers, Receiver Operating Characteristic (ROC) curve analysis was performed. Additionally, univariate and multivariate Cox regression models were utilized to identify factors affecting survival duration. All statistical analyses were conducted using SPSS (IBM SPSS Statistics for Windows, Version 22.0 (IBM Corp., Armonk, NY, USA)), with *p*-values < 0.05 considered statistically significant.

## 3. Results

In the present study, patients with confirmed ALK positivity in the classification based on NSCLC molecular subtypes within the specified time period were included in the study group, while patients with NSCLC without driver mutations were included in the control group. The clinical and demographic characteristics of the patients are presented in Table 1. While 81.4% of patients were male and 18.6% were female, the mean age at diagnosis and body mass index (BMI) were 63 ± 9.87 and 24.91 ± 4.66, respectively. Furthermore, 26.74% of patients were categorized as non-smokers, while 28.49% had a smoking history of less than 43 pack-years and 44.7% had a smoking history of at least 43 pack-years. The Eastern Cooperative Oncology Group (ECOG) performance status was evaluated, with 59.88% of patients having an ECOG score of 0, 35.47% an ECOG score of 1, and 4.65% an ECOG score of 2. Furthermore, the study revealed that 59.76% of patients had adenocarcinoma, 26.63% had squamous cell carcinoma, 1.78% had adenosquamous carcinoma, 10.06% had tumors not otherwise specified (NOS), and 1.78% had other tumor types. The primary tumor sites were determined as 67.44% right lung, 31.4% left lung, and 1.16% bilateral.

Furthermore, the temporal sequence of metastasis in patients was determined as follows: metastases were de novo in 76.92% of cases and metachronous in 23.08%. Rare metastases (thyroid, skin, small intestine, pancreas, eye, and kidney) were observed in 3.49% of patients, 34.3% had contralateral lung metastasis, 41.28% had bone metastasis, 20.93% had liver metastasis, 23.26% had adrenal metastasis, and 30.23% had brain metastasis. Furthermore, 7.56% of patients underwent primary surgery, and 47.09% (n: 81) were determined to be ALK-positive. Furthermore, 6.98% of patients received adjuvant therapy. The initial therapeutic approach adopted was Carboplatin + Paclitaxel in 47.88% (n = 79) of cases and Cisplatin + Gemcitabine in 12.12% (n = 20). It was observed that progression occurred in 95.93% (n = 165) of cases. The median time to first progression was 6.65 months (±6.57). The site of progression was observed in 60.84% of cases in the primary tumor area, 16.27% in the contralateral lung, and 12.65% as bone metastases. The final analysis revealed that 27.91% of patients were alive, while 72.09% had died. A comprehensive array of diagnostic procedures was conducted to exclude the possibility of acute infection, encompassing complete blood count, sputum culture, C-reactive protein testing, blood culture, sedimentation rate assessment, and procalcitonin quantification. No infection was detected in any of these results, and the patients were included in the study. Patients with infection detected in these results or with missing data were excluded from the study (Table 1).

Following a comprehensive analysis of the available data, it was determined that intergroup performance status (*p* = 0.055), primary tumor site (*p* = 0.191), timing of metastasis (*p* = 0.215), metastasis to the contralateral lung (*p* = 0.801), rare metastasis (*p* = 0.129), bone metastasis (*p* = 0.861), liver metastasis (*p* = 0.253), age at diagnosis (*p* = 0.109), BMI (*p* = 0.879), and progression (*p* = 0.256) were similar.

In the ALK-positive group, female gender (*p* = 0.002), non-smoking status (*p* = 0.001), adenocarcinoma histopathology (*p* = 0.001), the presence of brain metastases (*p* = 0.001), and progression most commonly at the primary tumor site and brain metastases (*p* = 0.008) were more frequent than in the ALK-negative group. Post first-line treatment PFS was higher in the ALK-negative group (5.17 months (95% CI: 3–9.4); 3.77 months (95% CI: 2–6.53); *p* = 0.015).

**Figure 2:**
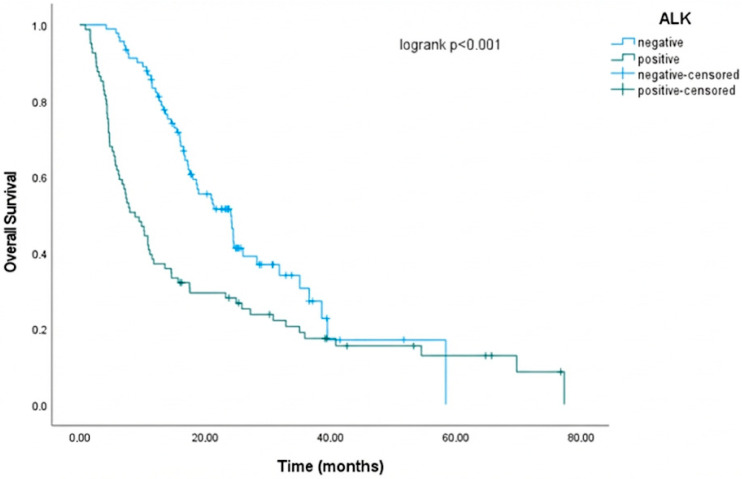
Survival analysis of ALK positivity performed using the Kaplan–Meier method.

In ALK-negative patients, the mOS was 24.1 months (95% CI: 18.503–29.697), while in ALK-positive patients, the mOS was 8.833 months (95% CI: 5.861–11.806; *p* < 0.001). The 2-year survival rate was 49.8% in the ALK-negative group and 28.1% in the ALK-positive group. This finding lends further support to the prevailing hypothesis that ALK positivity is associated with a poor prognosis (Table 2 and Figure 2).

**Figure 3:**
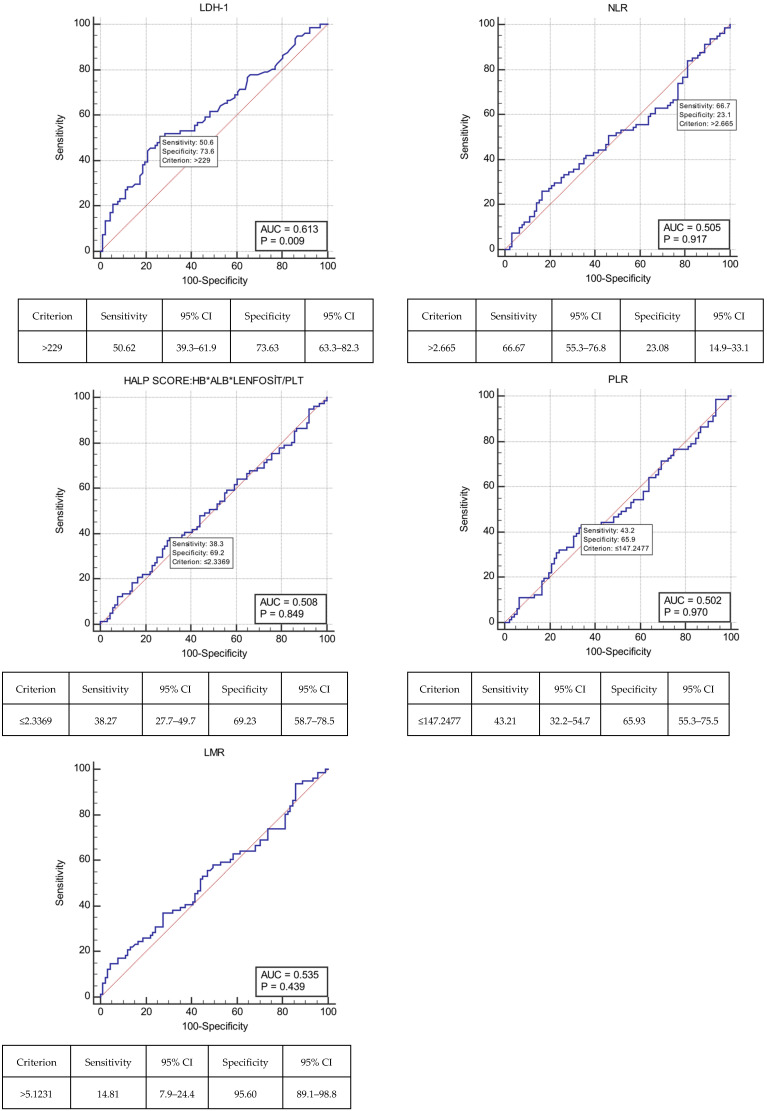
ROC curve analysis of lactate dehydrogenase (LDH); neutrophil/lymphocyte ratio (NLR); HALP (hemoglobin, albumin, lenfosit, and platelet) score; platelet/lymphocyte ratio (PLR); lymphocyte/monocyte ratio (LMR).

A comprehensive set of hematological and biochemical blood test results was obtained at the time of metastasis and meticulously recorded for further analysis. The incorporation of these results into established formulae enabled the calculation of prognostic markers, defined in the literature as indicators of systemic inflammation and nutrition. As these prognostic markers did not have predetermined cut-off values documented in the literature, cut-off values were determined using ROC curve analysis (LDH: ≤229; NLR: ≤2.665; HALP score: ≤2.3369; PLR: ≤147.2477; LMR: ≤5.1231) (Figure 3).

When grouped by ALK status, systemic inflammation and nutritional markers (LDH-1, NLR, HALP score, PLR, and LMR) calculated from blood results obtained at the time of metastasis were analyzed to detect bone, adrenal, and brain metastasis. The results showed no statistically significant difference.

Analysis of LDH levels relative to the defined cut-off value yielded statistically significant results for predicting metastasis to the contralateral lung (*p* = 0.032) and liver (*p* = 0.007). LDH levels were lower (≤229) in patients with metastasis to the contralateral lung, whereas they were higher (>229) in patients with metastasis to the liver. No significant differences were observed in other prognostic variables (NLR, HALP score, PLR, and LMR) between patients with metastasis to the contralateral lung and liver (Table 3).

Univariate and multivariate Cox regression analyses were performed on variables including metastasis site and systemic inflammation markers in patients with NSCLC. In the univariate analysis, the following variables were found to be statistically significant prognostic factors for survival: age at diagnosis, timing of metastasis, presence of liver metastasis, and the inflammation marker NLR. The timing of metastasis (de novo vs. metachronous) and the presence of liver metastasis were confirmed as independent predictors in the multivariate model. It was determined that the variables included in the univariate analysis would not be included in the final model, in accordance with the principle of ensuring at least 10 events per variable in the study. This finding acknowledges the limitations of retrospective assessment of systemic inflammation and nutritional markers, while emphasizing that the presence of liver metastasis is a decisive prognostic factor. In the multivariate analysis, the presence of liver metastasis was identified as an independent predictor of poor prognosis (HR = 1.618; 95% CI: 1.050–2.494; *p* = 0.029) (Table 4).

A comprehensive review of culture results, radiological examinations within the hospital imaging system, and retrospective file records was conducted to exclude the possibility of concomitant infection. The inclusion and exclusion criteria for the study group and control group were meticulously delineated, and the patient selection process is presented in the Strobe flow diagram (Figure 1).

In this study, patients with confirmed ALK positivity were included in the study group, while NSCLC patients without driver mutations were included in the control group. The prognostic effects of patients’ metastasis site and systemic inflammation markers for these metastasis sites were analyzed. In accordance with the extant literature, female gender, absence of a history of smoking, adenocarcinoma histopathology, and brain metastasis were more prevalent in the ALK-positive group. The two year survival rate was 49.8% in the ALK-negative group and 28.1% in the ALK-positive group. This finding lends further support to the poor prognosis associated with ALK positivity. The findings of this study demonstrated that systemic inflammation and nutritional markers (NLR, HALP score, PLR, and LMR), when analyzed in conjunction with LDH, did not serve as prognostic indicators in determining the metastasis site in NSCLC.

In the subgroup analysis of ALK-positive patients, the mOS was 7.37 ± 2.31 months for those who received ALK TKIs, compared with 10.30 ± 2.05 months for those who did not. The difference in survival times between the two groups was not statistically significant. Furthermore, no statistically significant difference was determined between ALK TKI treatment status and the presence of brain metastases.

## 4. Discussion

The present retrospective analysis employed RWD from an oncology center. The metastatic patterns and survival of ALK-positive NSCLC were examined by comparing them with a control group of NSCLC patients without driver mutations. The study data were analyzed in two distinct ways. Firstly, in ALK-positive NSCLC patients, we confirmed—in accordance with the extant literature—that female gender, non-smoking status, adenocarcinoma histopathology, presence of brain metastases, and progression most commonly occurring in the primary tumor site and brain metastases were more frequent compared to the ALK-negative group. Secondly, the mOS was 24.1 months (±2.855 months; 95% CI: 18.503–29.697) in ALK-negative patients and 8.833 months (±1.517 months; 95% CI: 5.861–11.806; *p* < 0.001) in ALK-positive patients. Pivotal studies, including PROFILE 1014 and ALEX, have established that access to ALK TKIs and pemetrexed-based regimens significantly extends survival for these patients. Consequently, the shorter overall survival observed in the real-world cohort compared with the major trials is likely attributable to the limited availability and utilization of life-extending agents in the first-line setting due to local reimbursement policies during the study period [11,23].

Unexpectedly, the present analysis revealed no statistically significant differences between patients in terms of overall survival or incidence of central nervous system progression, whether they were or were not treated with an ALK-TKIs. Large-scale trials show that ALK inhibitors improve survival, but our findings probably reflect restricted access to the drugs during the study period. Many in the treatment group may have received TKIs in later lines or for shorter times due to reimbursement constraints, reducing the potential survival advantage seen in trials.

The 2-year survival rate was 49.8% in the ALK-negative group and 28.1% in the ALK-positive group. This finding lends further support to the prevailing hypothesis that ALK positivity is associated with a poor prognosis. This outcome has been ascribed to the elevated prevalence of brain metastases within the ALK-positive patient cohort and the observation that progression in this patient group often occurs in conjunction with brain metastases.

Finally, systemic inflammation and nutritional markers (LDH, NLR, HALP score, PLR, and LMR) were calculated using hematological and biochemical blood test results obtained at the time of metastasis. When patients were grouped by ALK status, systemic inflammation and nutritional markers (NLR, HALP score, PLR, and LMR) showed no significant differences in detecting bone metastasis, adrenal metastasis, or brain metastasis. The analysis, based on LDH levels at a defined cut-off value, demonstrated the ability to statistically predict metastasis to the contralateral lung and liver. LDH values were lower (≤229) in patients with contralateral lung metastasis; conversely, they were higher (>229) in patients with liver metastasis. It was established that other prognostic variables (NLR, HALP score, PLR, and LMR) showed no significant difference between patients with and without contralateral lung and liver metastasis. Based on the findings, it can be concluded that systemic inflammation and nutritional markers (NLR, HALP score, PLR, and LMR) derived from blood test results at the time of metastasis are not useful for predicting the site of metastasis. The findings from this study are valid for all patients diagnosed with NSCLC, regardless of the specific driver mutation.

Cancer cells (Ewing) that reach the organ via blood flow can grow when they encounter a suitable microenvironment (Paget). It is an essential prerequisite for the survival of a cancer cell that a pre-metastatic niche (soil) is present. This interaction is perpetuated by molecular signaling [21]. In this study, the most prevalent form of metastasis was bone, accounting for 41.28% of cases. Consequently, denosumab treatment was initiated. In descending order of frequency, the following metastases were observed: 34.3% had metastasis to the contralateral lung, 30.23% had brain metastasis, 23.26% had adrenal metastasis, 20.93% had liver metastasis, and 3.49% had rare metastasis (thyroid, skin, small intestine, pancreas, orbita, and kidney). De novo metastases were observed in 76.92% of patients. Similarly, gene rearrangements, such as ALK and ROS1, have been associated with unusual metastasis patterns. These unusual metastasis patterns include, but are not limited to, ovarian or splenic metastases [32].

Given the high probability of metastasis in patients with NSCLC, research has been undertaken to elucidate the etiology of this condition. The Nuclear Factor-κB Ligand Receptor Activator (RANKL/RANK) signaling pathway is pivotal to bone remodeling and the pathogenesis of metastasis. Denosumab, a drug that targets RANKL, is the standard treatment for cases of bone metastasis. Consequently, by impeding the signaling pathway, the destruction of bone that results from osteocarcinogenesis is averted [33]. It is evident that RANKL exerts its influence not only on osteoclasts but also on the tumor microenvironment. Furthermore, endothelial cells express RANK, thereby increasing vascular permeability and angiogenesis. This process is known as extravasation and metastasis. The suppression of this mechanism through anti-RANKL therapies has also been shown to inhibit the development of distant metastasis [34].

In accordance with the extant literature, males were more prevalent (81.4% of cases), while females accounted for 18.6% of cases. The mean BMI at the time of metastasis was 24.91 ± 4.66. This finding indicated that patients were in the precatabolic phase and that inflammatory markers would not influence the outcome when evaluating their prognostic power. The risk of lung cancer increases with the duration of smoking. Although it is evident in younger demographics, its prevalence increases after the age of 40 [35]. This study revealed that 44.7% of patients had a smoking history of ≥43 pack-years. This finding lends further support to the prevailing hypothesis that an elevated smoking rate is associated with an increased risk of developing NSCLC. In accordance with the extant literature [3], 59.76% of patients exhibited adenocarcinoma, 26.63% exhibited squamous cell carcinoma, 1.78% exhibited adenosquamous carcinoma, and 10.06% exhibited NOS (not otherwise specified) tumor types.

Another clinical study focusing on the pathogenesis of metastasis found that the expression of the L1 cell adhesion molecule (L1CAM), which plays a role in epithelial–mesenchymal transition (EMT), occurred in 25% of squamous cell carcinomas and 24% of adenocarcinomas. It was thought that L1CAM was associated with the development of metastasis and blood vessel invasion (*p* < 0.05). In a multivariate analysis that included tumor grade, pT, pN, and metastasis status, L1CAM was found to be an independent variable for survival. L1CAM levels were found to be correlated with E-cadherin and associated with Slug, β-catenin, and Vimentin (*p* <0.05). E-cadherin expression was higher in the tumor center, while L1CAM expression was higher in the tumor microenvironment. Matrigel invasion was high in the group with high L1CAM expression, decreasing after anti-L1CAM application. L1CAM expression was found to increase in NSCLC patients with a higher frequency of metastasis and vascular tropism, thereby inducing EMT. It is thought that metastasis occurs due to increased cell motility. Following EMT, cancer cells escape the confines of the tumor, migrate, and acquire an aggressive fibroblastic phenotype. This study also found that L1CAM expression may be heterogeneous due to tumor heterogeneity. The mechanism by which L1CAM levels increase remains unclear. L1CAM levels were found to be prognostic for mOS and PFS [36].

A recent study investigating the prognostic value of L1CAM in the literature hypothesized that long noncoding RNAs (lncRNAs) play a significant role in tumor formation. LncRNAs have been shown to competitively bind to miRNAs involved in cancer pathogenesis. These proteins play a crucial role in regulating gene expression. In patients with NSCLC, the target gene of microRNAs (miRs) 6783-3p and 1343-3p, L1CAM, was found to be upregulated and showed a positive correlation with LINC02323 expression. This result supports the conclusion that LINC02323 inhibits both miR-6783-3p and miR-1343-3p. The molecular mechanisms identified have been proposed as potential prognostic markers and therapeutic targets in NSCLC [37]. In patients with NSCLC who have undergone surgery for brain metastases, high L1CAM expression has been demonstrated to be a poor prognostic factor for mOS. This phenomenon has been associated with alterations in the tumor microenvironment. It is anticipated that clinical and preclinical studies evaluating the therapeutic efficacy of L1CAM blockade in this patient group will prove to be instructive [38].

When these results are evaluated, it is hypothesized that L1CAM expression contributes to organotropism in metastasis. As preclinical and clinical studies conducted at the molecular level continue to evaluate its effect on the tumor microenvironment and the metastasis process, updates in this field will increase. Consequently, the number of targeted therapies will increase. Despite increased survival data for TKI treatments in recent clinical studies, this study demonstrated that the overall survival of ALK-positive patients was lower than that of patients without driver mutations. This outcome is incongruent with the mounting treatment responses documented in the extant literature; nevertheless, the elevated incidence of brain metastasis in cases of ALK positivity, coupled with the recurrent occurrence of progression in the brain, elucidates the diminished survival data observed in this study, which stands in contrast to the findings reported in the literature. The development of NSCLC is a process that unfolds over years and is characterized by the accumulation of epigenetic and genetic alterations. The prognosis for this condition is poor, primarily due to its high potential for distant metastasis. It is noteworthy that brain metastasis is observed in 10% of newly diagnosed NSCLC cases. Although brain metastases are observed in a wide range of cancer types, NSCLC (40–50%) has been demonstrated to be the most prevalent primary tumor location. Consequently, brain imaging is imperative for staging [39,40].

Recent studies consistently show that oncogene-addicted NSCLCs exhibit different metastatic tropisms compared to those without driver mutations [41,42,43,44,45]. Our study, in agreement with 2023 findings, confirms a unique pattern of ALK-rearranged tumor spread, supporting a biological link between specific drivers and metastasis.

The primary step in the management of brain metastases in advanced NSCLC is evaluating the feasibility of local treatments, which may include surgery or stereotactic radiosurgery (SRS). In instances where there is a threat of mass-related herniation or where curative treatment is planned in oligometastatic disease diagnosed with a solitary brain metastasis, surgical intervention is recommended [46,47]. Other treatment options encompass systemic chemotherapy and targeted therapies. Notwithstanding the advances in medical science and technology, the survival rate of these patients remains dismally low, typically spanning only a few years. A heightened propensity for brain metastasis has been observed in patients with this condition, particularly in those with specific genetic alterations, namely, EGFR and ALK mutations. In such cases, first-line local treatments have been superseded by newer-generation TKIs, which have been demonstrated to exhibit higher intracranial efficacy [48,49,50].

In ALK-positive patients who do not require urgent surgical intervention, systemic therapies are the preferred option [51]. The rationale behind this phenomenon is that, despite the robust extra-cranial activity, if the effective dose of treatment fails to reach the brain tissue in brain metastases, this tissue functions as a “refuge area” for tumor cells, thereby facilitating the progression of brain metastases. The advent of new-generation TKI treatments has enabled the preservation of brain cognitive functions, which are prone to deterioration following surgery and radiotherapy, for extended periods of time [52,53]. In patients with low ECOG performance, the optimal treatment strategy would be best supportive care (BSC), which includes the administration of steroids.

This study revealed no statistically significant differences between the groups formed according to ALK status in terms of performance status (*p* = 0.055), primary tumor location (*p* = 0.191), bone metastasis (*p* = 0.861), rare metastasis (*p* = 0.129), timing of metastasis (*p* = 0.215), metastasis to the opposite lung (*p* = 0.801), liver metastasis (*p* = 0.253), history of surgery (*p* = 0.944), stage (*p* = 0.447), age at diagnosis (*p* = 0.109), BMI (*p* = 0.879), and progression (*p* = 0.256). In the ALK-positive group, female gender (*p* = 0.002), non-smoking status (*p* = 0.001), adenocarcinoma histopathology (*p* = 0.001), the presence of brain metastasis (*p* = 0.001), and progression most commonly at the primary tumor site and in brain metastasis (*p* = 0.008) were more frequent than in the ALK-negative group. Post-first-line treatment PFS was higher in the ALK-negative group (5.17 months (95% CI: 3–9.4); 3.77 months (95% CI: 2–6.53); *p* = 0.015). This result is inconsistent with the extant literature. The high rate of brain metastases in patients and their most frequent progression may be implicated. Another possibility is that, due to the health payment conditions in our country, only alectinib could be used in first-line treatment, and lorlatinib, which has been shown to provide the greatest increase in survival [54], could not be administered. It is hypothesized that future studies incorporating updated results from current studies and revised payment conditions will yield improved survival outcomes.

The optimal cut-off values for the prognostic markers, determined to assess systemic inflammation status at the time of metastasis in patients (LDH: ≤229; NLR: ≤2.665; HALP score: ≤2.3369; PLR: ≤147.2477; LMR: ≤5.1231), were identified using ROC curve analysis. A comprehensive diagnostic approach, including various analytical tests, was employed to rule out alternative diagnoses. These included acute infection, complete blood count, sputum culture, C-reactive protein (CRP) test, blood culture, sedimentation rate, and procalcitonin tests. When grouped by ALK status, systemic inflammation markers (LDH, NLR, HALP score, PLR, and LMR) calculated from blood results obtained at the time of metastasis were analyzed for the detection of bone, adrenal, and brain metastasis. No significant differences were found. In a retrospective study of patients with NSCLC, a high HALP score was associated with improved PFS. Among the prognostic variables included in the multivariate analysis, only the HALP score was prognostic for PFS (HR: 0.539, 95% CI: 0.331–0.876, *p* = 0.013). In light of these findings, it was proposed that the test could serve as a prognostic marker; that is, a tool used to predict how patients will respond to treatment [27]. However, unlike previous studies, this investigation did not identify a significant association between the HALP score and patient prognosis or metastasis location.

The analysis based on LDH cut-off values demonstrated that LDH levels can predict metastasis to the contralateral lung (*p* = 0.032) and liver (*p* = 0.007). LDH levels were lower (≤229) in patients with contralateral lung metastasis; conversely, they were higher (>229) in patients with liver metastasis. No significant differences were observed in the other analyzed prognostic variables (NLR, HALP score, PLR, and LMR) according to the presence of contralateral lung and liver metastasis. A meta-analysis of 19 clinical studies involving patients with NSCLC revealed that elevated LDH levels were associated with reduced OS (HR = 1.19, 95% CI = 1.11–1.24, *p* < 0.001). Asymmetric funnel plots and the Egger test indicated a high potential for publication bias in the HR results for OS (*p* < 0.001) and PFS (*p* < 0.001). Research conducted with small sample sizes has been shown to carry an elevated risk [28]. As emphasized in the results of this meta-analysis, negative findings should be clearly stated, and there should be no publication bias.

In the univariate analysis of prognostic markers in patients with NSCLC, age at diagnosis, timing of metastasis, presence of liver metastasis, and the NLR level among inflammation markers were found to be significant prognostic variables for survival. A meta-analysis of 27 publications involving a total of 4298 patients diagnosed with NSCLC revealed that elevated NLR levels prior to treatment were associated with reduced OS (HR: 1.63, 95% CI: 1.43–1.84) and PFS (HR: 1.45, 95% CI: 1.28–1.66) [29]. This finding suggests that NLR levels may serve as a prognostic marker in patients with NSCLC. A significant correlation has been demonstrated between pretreatment NLR and PLR levels and the efficacy and prognosis of opioid treatment in patients with NSCLC receiving immune checkpoint inhibitor (ICI) therapy [30]. The result of this study indicates that the PLR value, analyzed as a prognostic marker, was not significant. The hypothesis was that increased patient numbers and multicenter studies would be beneficial. The current state of research does not yet allow for predicting survival in advanced NSCLC patients. However, this is a subject under ongoing research. It has been established that elevated PLR, high NLR, and low LMR at the commencement of treatment are significantly associated with poor OS [31].

These biomarkers represent a cost-effective, accessible method for analyzing systemic inflammation. The clinical value of easily accessible biomarkers is recognized by the majority of medical oncologists worldwide. The early classification of patients’ treatment response is enabled by these biomarkers. The presence of de novo metastasis and liver metastasis was found to be an independent predictor in the multivariate model. It was established that the variables included in the univariate analysis were not included in the final model, due to the principle of including at least 10 events per variable in the study. While acknowledging the limitations of retrospective assessment of systemic inflammation and nutritional markers, these results emphasize that the presence of liver metastasis is a decisive variable in poor prognosis. In the multivariate analysis, the presence of liver metastasis was identified as an independent predictor of poor prognosis (HR: 1.618; 95% CI: 1.050–2.494; *p* = 0.029). Patients with liver metastasis exhibited a 1.6 times higher risk of progression. The confidence interval was 1.050; since it was greater than 1, the result was deemed reliable.

The most significant limitations of our study are that it is a single-center experience and that the data were obtained from retrospective medical records. In the context of our study, it is hypothesized that had the patient group been administered the most current and highest-survival-rate TKI—rather than the TKI available due to payment conditions in our country—higher survival rates would have been achieved. As this study is retrospective, reimbursement policies and patient access to medications were subject to limitations during the period under investigation in our country. Consequently, the patients had to undergo a course of chemotherapy. This predicament is attributable to the constraints imposed by RWD, thereby elucidating the rationale behind the observed paucity in survival rates.

Consequently, future prospective studies evaluating prognostic inflammatory markers will demonstrate their prognostic value (NLR, PLR, CLR, SII, and SIRI). The exclusion criteria for this study included a history of a second malignancy, chronic rheumatic disease, and a different driver mutation. The exclusion criteria are confounding factors that may affect the results of prognostic inflammation markers. The absence of any definition of this phenomenon in the exclusion criteria of the relevant studies is a factor that adds value to the results of this study.

## 5. Conclusions

In this study, we confirmed that certain characteristics were more prevalent in ALK-positive NSCLC patients compared to the ALK-negative group. The mOS for patients with ALK-negative and ALK-positive diagnoses was 24.1 and 8.8 months, respectively (*p* < 0.001). This finding provides further evidence that ALK positivity is associated with a poor prognosis; in particular, these results suggest that ALK positivity was associated with a poor prognosis before the introduction of new-generation TKIs. When groups of patients were stratified by ALK status, systemic inflammation and nutritional markers (NLR, HALP score, PLR, and LMR) were not prognostic for bone, adrenal, or brain metastasis. This result may be attributed to the influence of multiple additional factors, driven by the increased inflammatory process at the metastasis stage. Brain metastasis is common in ALK-positive patients. Timing (de novo or metachronous) and liver metastasis can independently predict prognosis.

## Figures and Tables

**Table 1:** The clinical and demographic characteristics of non-small cell lung cancer.

Variables		N (%)
Gender	Male	140 (81.4)
	Female	32 (18.6)
Smoking history	No	46 (26.74)
	<43 packs year	49 (28.49)
	≥43 packs year	77 (44.77)
Eastern cooperative oncology group (ECOG) performance status	0	103 (59.88)
	1	61 (35.47)
	2	8 (4.65)
Histopathology	Adenocarcinoma	101 (59.76)
	Squamous cell carcinoma	45 (26.63)
	Adenosquamous	3 (1.78)
	Not otherwise specified (NOS)	17 (10.06)
	Others	3 (1.78)
Timing of metastasis	De novo metastasis	130 (76.92)
	Metacron metastasis	39 (23.08)
Rare metastasis	No	166 (96.51)
	Yes	6 (3.49)
Metastasis to the opposite lung	No	113 (65.7)
	Yes	59 (34.3)
Bone metastasis	No	101 (58.72)
	Yes	71 (41.28)
Liver metastasis	No	136 (79.07)
	Yes	36 (20.93)
Adrenal metastasis	No	132 (76.74)
	Yes	40 (23.26)
Brain metastasis	No	120 (69.77)
	Yes	52 (30.23)
ALK rearrangement (Anaplastic Lymphoma Kinase rearrangement)	Negative	91 (52.91)
	Positive	81 (47.09)
First-line systemic treatment	Cisplatin + Gemcitabine	20 (12.12)
	Carboplatin + Paclitaxel	79 (47.88)
	Cisplatin + Pemetrexed	7 (4.24)
	Alectinib	43 (26.06)
	Crizotinib	6 (3.64)
	Nivolumab	4 (2.42)
	Cisplatin + Etoposide	3 (1.82)
	Pembrolizumab	3 (1.82)
Progression 1	No	7 (4.07)
	Yes	165 (95.93)
Progression 1 location	Primary tumor progression	101 (60.84)
	Contralateral lung new metastasis	27 (16.27)
	Brain metastasis	8 (4.82)
	Bone metastasis	21 (12.65)
	Liver metastasis	4 (2.41)
	Adrenal metastasis	5 (3.01)
Second line systemic treatment	Cisplatin + Gemcitabine	23 (18.7)
	Carboplatin + Paclitaxel	8 (6.5)
	Cisplatin + Pemetrexed	4 (3.25)
	Alectinib	15 (12.2)
	Brigatinib	2 (1.63)
	Lorlatinib	9 (7.32)
	Nivolumab	62 (50.41)
Progression 2	No	20 (16.26)
	Yes	103 (83.74)
Living situation	Alive	48 (27.91)
	Exitus	124 (72.09)
Age at diagnosis (median (min–max))		63.2 (25.91–89.44)
BMI (kg/m^2^) (median (min–max))		24.58 (14.69–42.32)
PFS (median (min–max))		4.85 (0.07–45.4)
mOS (month)		16.633 ± 1.438 (95% CI = 13.815–19.452)
2 year survival		39.7%

BMI: body mass index; PFS: progression-free survival; min: minimum; max: maximum; mOS: median survival.

**Table 2:** A comparison of clinical and demographic characteristics according to ALK Rearrangement (anaplastic lymphoma kinase) status.

Variables		ALK Rearrangement (Anaplastik Lenfoma Kinase)		*p*
		Negative	Positive	
		N (%)	N (%)	
Gender	Male	82 (90.11)	58 (71.6)	0.002 *
	Female	9 (9.89)	23 (28.4)	
Smoking history	No	11 (12.09)	35 (43.21)	0.001 *
	<43 packs year	27 (29.67)	22 (27.16)	
	≥43 packs year	53 (58.24)	24 (29.63)	
Eastern cooperative oncology group (ECOG) performance status	0	55 (60.44)	48 (59.26)	0.055
	1	35 (38.46)	26 (32.1)	
	2	1 (1.1)	7 (8.64)	
Histopathology	Adenocarcinoma	40 (45.45)	61 (75.31)	0.001 *
	Squamous cell carcinoma	41 (46.59)	4 (4.94)	
	Adenosquamous	1 (1.14)	2 (2.47)	
	Not otherwise specified (NOS)	6 (6.82)	11 (13.58)	
	Other	0 (0)	3 (3.7)	
Timing of metastasis	De novo metastasis	65 (71.43)	65 (83.33)	0.215
	Metacron metastasis	26 (28.57)	13 (16.67)	
Rare metastasis	No	86 (94.51)	80 (98.77)	0.129
	Yes	5 (5.49)	1 (1.23)	
Metastasis to the opposite lung	No	59 (64.84)	54 (66.67)	0.801
	Yes	32 (35.16)	27 (33.33)	
Bone metastasis	No	54 (59.34)	47 (58.02)	0.861
	Yes	37 (40.66)	34 (41.98)	
Liver metastasis	No	75 (82.42)	61 (75.31)	0.253
	Yes	16 (17.58)	20 (24.69)	
Adrenal metastasis	No	67 (73.63)	65 (80.25)	0.305
	Yes	24 (26.37)	16 (19.75)	
Brain metastasis	No	74 (81.32)	46 (56.79)	0.001 *
	Yes	17 (18.68)	35 (43.21)	
Stage at diagnosis	1	2 (2.2)	4 (4.94)	0.447
	2	5 (5.49)	2 (2.47)	
	3	21 (23.08)	14 (17.28)	
	4	63 (69.23)	61 (75.31)	
Programmed death ligand 1 (PDL1)	0	25 (27.47)	6 (7.41)	0.001 *
	1–49	27 (29.67)	8 (9.88)	
	≥50	39 (42.86)	16 (19.75)	
	Unknown	0 (0)	51 (62.96)	
First-line systemic treatment	Cisplatin + Gemcitabine	17 (18.68)	3 (4.05)	-
	Carboplatin + Paclitaxel	63 (69.23)	16 (21.62)	
	Cisplatin + Pemetrexed	3 (3.3)	4 (5.41)	
	Alectinib	0 (0)	43 (58.11)	
	Crizotinib	0 (0)	6 (8.11)	
	Nivolumab	4 (4.4)	0 (0)	
	Cisplatin + Etoposide	1 (1.1)	2 (2.7)	
	Pembrolizumab	3 (3.3)	0 (0)	
Progression 1	No	2 (2.2)	5 (6.17)	0.256
	Yes	89 (97.8)	76 (93.83)	
Progression 1 location	Primary tumor progression	48 (53.93)	53 (68.83)	0.008 *
	Contralateral lung new metastasis	18 (20.22)	9 (11.69)	
	Brain metastasis	1 (1.12)	7 (9.09)	
	Bone metastasis	14 (15.73)	7 (9.09)	
	Liver metastasis	4 (4.49)	0 (0)	
	Adrenal metastasis	4 (4.49)	1 (1.3)	
Age at diagnosis	(mean ± SS/median (min–max))	64.27 ± 7.5/63.68 (59.59–69.77)	61.57 ± 11.87/62.75 (56.02–67.35)	0.109
BMI (kg/m^2^)	(median (min–max))	24.49 (21.33–27.61)	24.58 (21.88–26.78)	0.879
PFS (month)	(median (min–max))	5.17 (3–9.4)	3.77 (2–6.53)	0.015 *
mOS (month)		24.1 ± 2.855 (95% CI: 18.503–29.697)	8.833 ± 1.517 (95% CI: 5.861–11.806; *p* < 0.001)	<0.001

BMI: body mass index; PFS: progression-free survival; min: minimum; max: maximum; mOS: median overall survival; Mann–Whitney U test; survival analysis; statistically significant results (*p* < 0.05) are indicated with a (*) sign next to the *p* value.

**Table 3:** Association of Anaplastic Lymphoma Kinase (ALK) rearrangement status, systemic inflammation, and nutritional markers with metastatic site distribution in NSCLC patients.

Variables	Metastasis to the Opposite Lung		*p*	Bone Metastasis		*p*	Liver Metastasis		*p*
	ALK-Negative	ALK-Positive		ALK-Negative	ALK-Positive		ALK-Negative	ALK-Positive	
LDH median (Q1–Q3)	219 (169–296)	187 (150–254)	0.032 *	200 (156–279)	210 (169–274)	0.370	194.5 (159–273)	247 (183–352.5)	0.007 *
NLR median (Q1–Q3)	3.59 (2.55–5.4)	3.82 (2.69–4.96)	0.735	3.47 (2.55–4.77)	3.68 (2.73–5.82)	0.161	3.47 (2.54–5.39)	3.93 (3.02–4.87)	0.380
HALP score median (Q1–Q3)	2.88 (1.99–4.1)	2.98 (1.7–4.66)	0.973	2.97 (1.99–4.66)	2.77 (1.85–3.94)	0.291	2.95 (2.02–4.48)	2.79 (1.54–4.25)	0.783
PLR median (Q1–Q3)	173.05 (126.41–239.46)	172.96 (114.9–250.67)	0.965	170.75 (109.33–229.61)	179.75 (137.91–271.43)	0.100	170.14 (124.81–244.42)	192.06 (125.3–234.6)	0.786
LMR median (Q1–Q3)	2.67 (1.86–4)	2.7 (2.02–3.71)	0.947	2.78 (2.04–3.96)	2.48 (1.81–3.93)	0.189	2.69 (1.92–3.95)	2.62 (2.03–3.6)	0.743
Variables	Adrenal metastasis		*p*	Brain metastasis		*p*			
	ALK-negative	ALK-positive		ALK-negative	ALK-positive				
LDH median (Q1–Q3)	203.5 (159–279.5)	217 (174–273)	0.491	195.5 (158.5–272)	224.5 (173.5–300)	0.115			
NLR median (Q1–Q3)	3.51 (2.61–5.35)	4 (2.71–5.15)	0.718	3.51 (2.64–5.11)	3.97 (2.59–6.56)	0.490			
HALP score median (Q1–Q3)	2.96 (1.86–4.49)	2.88 (2.02–4.04)	0.928	2.86 (1.82–3.98)	3.05 (2.2–4.85)	0.154			
PLR median (Q1–Q3)	165.98 (124.81–242.12)	188.12 (128.51–240.14)	0.698	173.25 (126.75–245.14)	167.13 (116.05–234.04)	0.594			
LMR median (Q1–Q3)	2.69 (1.92–3.95)	2.69 (2–3.75)	0.731	2.66 (2.02–3.49)	3.29 (1.71–4.34)	0.376			

Anaplastic Lymphoma Kinase Rearrangement (ALK); lactate dehydrogenase (LDH); neutrophil/lymphocyte ratio (NLR); HALP (hemoglobin, albumin, lenfosit, and platelet) score; platelet/lymphocyte ratio (PLR); lymphocyte/monocyte ratio (LMR). The data are presented as median interquartile range (Q1–Q3). *p*-values (<0.05 *) were determined through the implementation of the Mann–Whitney U test.

**Table 4:** Univariate and multivariate Cox regression analysis of metastasis site and systemic inflammation markers for survival in NSCLC.

Variables		Univariate Analysis		Multivariate Analysis	
		HR (95% CI)	*p*	HR (95% CI)	*p*
Timing of metastasis	De novo metastasis	1.939 (1.235–3.044)	0.004 *	1.755 (1.100–2.801)	0.018 *
	Metacron metastasis	1 (reference)			
Metastasis to the opposite lung	No	1 (reference)			
	Yes	0.794 (0.544–1.159)	0.233		
Bone metastasis	No	1 (reference)			
	Yes	1.156 (0.809–1.653)	0.426		
Liver metastasis	No	1 (reference)			
	Yes	1.764 (1.164–2.675)	0.008 *	1.618 (1.050–2.494)	0.029 *
Adrenal metastasis	No	1 (reference)			
	Yes	0.873 (0.566–1.347)	0.540		
Brain metastasis	No	1 (reference)			
	Yes	1.328 (0.912–1.933)	0.139		
LDH	≤229	1 (reference)			
	>229	1.266 (0.881–1.818)	0.203		
NLR	≤2.665	1 (reference)			
	>2.665	1.515 (1.007–2.277)	0.046 *		
LMR	≤5.1231	1 (reference)			
	>5.1231	1.175 (0.669–2.062)	0.574		
HALP score	≤2.3369	1.194 (0.821–1.736)	0.354		
	>2.3369	1 (reference)			
PLR	≤147.2477	0.812 (0.563–1.171)	0.265		
	>147.2477	1 (reference)			

Hazard ratio (HR); confidence interval (CI); lactate dehydrogenase (LDH); neutrophil/lymphocyte ratio (NLR); HALP (hemoglobin, albumin, lenfosit, and platelet) score; platelet/lymphocyte ratio (PLR); lymphocyte/monocyte ratio (LMR). Statistically significant results (*p* < 0.05) are indicated with a (*) sign next to the *p* value.

## Data Availability

The data underlying this article will be shared upon reasonable request to the corresponding author.

## Abbreviations

The following abbreviations are used in this manuscript:

NSCLC	Non-small cell lung cancer.
EGFR	Epidermal growth factor receptor.
ALK	Anaplastic lymphoma kinase rearrangement.
ROS1	ROS proto-oncogene 1.
PDL1	Programmed death ligand 1.
EML4-ALK	Echinoderm microtubule-associated protein-like 4 fusion oncogene–anaplastic lymphoma kinase.
PET	Positron emission tomography.
MRI	Magnetic resonance imaging.
IHC	Immunohistochemistry.
FISH	Fluorescence in situ hybridization.
NGS	Next-generation sequencing.
PCR	Reverse transcription polymerase chain reaction.
mOS	Median overall survival.
PFS	Progression-free survival.
TKI	Tyrosine kinase inhibitor.
RWD	Real-world data.
HALP score	Hemoglobin, albumin, lymphocyte, and platelet score.
NLR	Neutrophil-to-lymphocyte ratio.
PLR	Platelet-to-lymphocyte ratio.
LMR	Lymphocyte-to-monocyte ratio.
LDH	Lactate dehydrogenase.
ROC	Receiver operating characteristic.
RANKL/RANK signaling pathway	Nuclear Factor-κB ligand receptor activator signaling pathway.
NOS	Not otherwise specified.
L1CAM	L1 cell adhesion molecule.
EMT	Epithelial–mesenchymal transition.
lncRNAs	Long noncoding RNAs.
miRNAs	MicroRNAs.
SRS	Stereotactic radiosurgery.
TKIs	Tyrosine kinase inhibitors.
BSC	Best supportive care.
CRP	C-reactive protein.
ICI	Immune checkpoint inhibitor.
